# Horticulture-based Interventions to Enhance Health and Wellbeing of People Living With Dementia in the Community

**DOI:** 10.1093/geroni/igab046.2443

**Published:** 2021-12-17

**Authors:** Theresa Scott, Ying-Ling Jao, Kristen Tulloch, Yates Eloise, Nancy Pachana

**Affiliations:** 1 The University of Queensland, St Lucia, Queensland, Australia; 2 Pennsylvania State University, University Park, Pennsylvania, United States

## Abstract

The majority of people living with dementia in the early and middle stages are cared for at home by family caregivers. Participation in meaningful activities is important for good quality of life. Recreation based on horticulture is beneficial for people living with dementia in residential settings, yet evidence within community settings is less clear. The aim of this research was to examine the existing evidence for the impact of using contact with nature, gardens and plants to enhance well-being of people living with dementia in the community. Our secondary aim was to explore the outcome domains and instruments that were employed in the existing research studies, to inform future research efforts and guide clinical practice. A systematic search was conducted covering several databases and gray literature. Original studies that examined group or individual horticulture-based activities or interventions were included. Of 2127 articles identified through searching, 10 were selected for full review. The findings reveal that horticulture-based intervention showed positive impacts on food intake, social interaction, and well-being in older adults with dementia. Some evidence shows that horticulture-based activities may alleviate stressful symptoms associated with living with dementia. Future research may further evaluate the effect of the interventions on cognitive function, physical function, and behavioral symptoms in a more rigorous intervention design.

